# Graphene-coated materials using silica particles as a framework for highly efficient removal of aromatic pollutants in water

**DOI:** 10.1038/srep11641

**Published:** 2015-06-29

**Authors:** Kaijie Yang, Baoliang Chen, Lizhong Zhu

**Affiliations:** 1 Department of Environmental Science, Zhejiang University, Hangzhou 310058, China; 2 Zhejiang Provincial Key Laboratory of Organic Pollution Process and Control, Hangzhou 310058, China

## Abstract

The substantial aggregation of pristine graphene nanosheets decreases its powerful adsorption capacity and diminishes its practical applications. To overcome this shortcoming, graphene-coated materials (GCMs) were prepared by loading graphene onto silica nanoparticles (SiO_2_). With the support of SiO_2_, the stacked interlamination of graphene was held open to expose the powerful adsorption sites in the interlayers. The adsorption of phenanthrene, a model aromatic pollutant, onto the loaded graphene nanosheets increased up to 100 fold compared with pristine graphene at the same level. The adsorption of GCMs increased with the loading amount of the graphene nanosheets and dramatically decreased with the introduction of oxygen-containing groups in the graphene nanosheets. The highly hydrophobic effect and the strong π-π stacking interactions of the exposed graphene nanosheets contributed to their superior adsorption of GCMs. An unusual GCM peak adsorption coefficient (*K*_d_) was observed with the increase in sorbate concentration. The sorbate concentration at peak *K*_d_ shifted to lower values for the reduced graphene oxide and graphene relative to the graphene oxide. Therefore, the replacement of water nanodroplets attached to the graphene nanosheets through weak non-hydrogen bonding with phenanthrene molecules via strong π-π stacking interactions is hypothesized to be an additional adsorption mechanism for GCMs.

Graphene is a novel 2D nanomaterial that is only one atom thick^1^. As a new class of carbon nanomaterials, its discovery has aroused increasing attention in chemical and environmental applications because of its easy chemical decoration^2^,^3^,^4^ and excellent performance in pollutant enrichment^5^,^6^,^7^,^8^. Graphene possess a large theoretical surface area (2630 m^2^/g)^9^, an electron-rich π-system^10^ and highly hydrophobic surface^2^,^3^ which make it a potential revolutionary adsorbent for environmental pollutant management^11^,^12^,^13^,^14^,^15^,^16^. CVD graphene is often used for functionalization and chemical applications^2^,^3^, while reduced graphene oxides are mostly exploited for chemical and environmental applications^4^,^15^,^16^. The graphene adsorption sites available for aromatic organic pollutants are mainly supplied by the hydrophobic carbon conjunction surface for π-π interactions^17^,^18^,^19^,^20^. Furthermore, defects, step-edges and groove/wrinkle areas are considered high-surface-energy adsorption sites and are preferentially occupied by external molecules^17^,^19^,^21^. However, pristine graphene nanosheets aggregate heavily in water due to large-area π-π interactions and strong van der Waals interactions between the graphene layers^10^,^17^,^19^, which inhibits the material’s powerful adsorption capacity^5^,^22^,^23^. This stacked aggregation limits access to many potential adsorption sites in the interlayers that are suppressed by confined water between the graphene sheets^24^. The use of graphene nanosheets as a highly effective adsorbent for persistent organic pollutants in water has not been explored.

The surface functional groups of graphene, such as oxygen-containing groups (OCGs), regulate the availability of adsorption sites and the dispersity of the graphene nanosheets in water^17^,^18^,^23^,^25^,^26^. Graphene oxide, the precursor of pristine graphene, contains many OCGs (including hydroxyl groups, carboxyl groups, and epoxy groups)^4^. Consequently, graphene oxide is easily dissolved in aqueous solution and fully expresses its adsorption sites^17^,^26^. However, the hydrophilic surfaces and weak π electron structure of graphene oxide result in a weak affinity for aromatic organic pollutants^4^,^6^,^17^,^18^. Graphene oxide reduction is the primary method used to prepare pristine graphene^8^,^27^. The recovery of the hydrophobic character and π electron structure of graphene significantly enhances its adsorption capabilities^15^,^17^,^18^,^21^,^27^, although the aggregation of graphene reduces the number of potential adsorption sites. To overcome aggregation, Zhao *et al*. successfully obtained well-dissolved sulfonated graphene by introducing sulfonic acid groups to the graphene surface^5^. This sulfonated graphene displayed extremely high adsorption capabilities for persistent aromatic pollutants because the adsorption sites that were initially hidden in the interlaminations of pristine graphene were exposed on the dissolved sulfonated graphene^5^. The hydrophobic nature of graphene limits its dispersion in water, whereas dispersed sulfonated graphene is difficult to separate from treated water. The dispersibility and separation of graphene nanosheets are equally critical to its environmental applications.

One promising strategy is to develop graphene-based materials by loading graphene nanosheets onto low-cost substrates^19^,^22^, which could allow the full expression of the graphene adsorption sites while simultaneously being easily separated from water. Recently, several studies have focused on the combination of graphene and SiO_2_. This type of composition has been used in solid-phase extraction material^28^, liquid chromatographic packing^29^ and sorbents for organophosphorus pesticides in water^22^. In our previous study^19^, a stable monolayer and several-layer graphene nanosheets in water was fabricated by a facile method via loading on nanosilica substrates, and this material displayed a higher adsorption capacity than other sorbents (such as graphite carbon, activated carbon, and pristine graphene) for aromatic pollutants. However, the maximum loading amount of graphene on SiO_2_ and the effects of graphene loading percentage on their adsorption performance need to be investigated for practical fabrication and environmental applications; and the true potential of the adsorption mechanisms of the loaded graphene nanosheets need further elucidation to consider the effects of OCGs on the graphene-water interface. Silica (SiO_2_) is an ideal framework material for mitigating graphene aggregation because it is an inexpensive, nontoxic and widespread material that can be manufactured in megatons per year^30^,^31^. Furthermore, because of the widespread presence of SiO_2_ in the environment, interactions between SiO_2_ and graphene are unavoidable when graphene is discharged into the environment. Thus, the adsorption capabilities and mechanisms of graphene-coated silica composites with different loadings and various OCG contents are worth further investigation.

The main objective of this study is to fabricate a highly efficient graphene-based adsorbent for organic pollutant management. To optimize practical performance of graphene materials in pollutant management, a series of GCMs were prepared by loading graphene oxide (GO), reduced graphene oxide (rGO) and graphene (G) nanosheets onto SiO_2_ particles with different loading percentages to probe the effects of graphene loading percentages and the OCG contents. The structures of SiO_2_, NH_2_-SiO_2_, pristine graphene and GCMs were characterized by elemental analysis, surface area, surface charge, FTIR, SEM and TEM to reveal the materials’ microscopic properties. Phenanthrene was selected as a model aromatic pollutant to determine the materials’ adsorption abilities and as a probe molecule to understand the interaction mechanisms at the graphene-water interface. In addition, the roles of attached water molecules on different graphene-water interfaces are discussed.

## Results and discussion

### Preparation and structural characterization of graphene-coated materials

**Figure 1:**
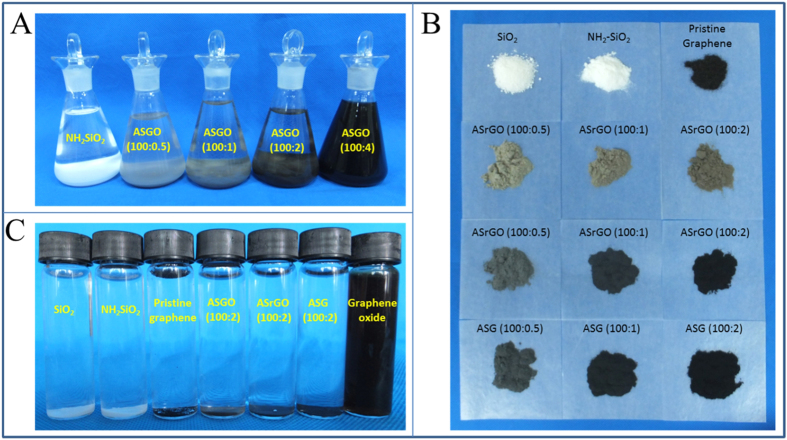
Digital images of experiment tests and resulting materials. (**A**) Maximum loading percentage test. (**B**) Graphene-coated materials. (**C**) Precipitation experiments in water.

**Figure 2:**
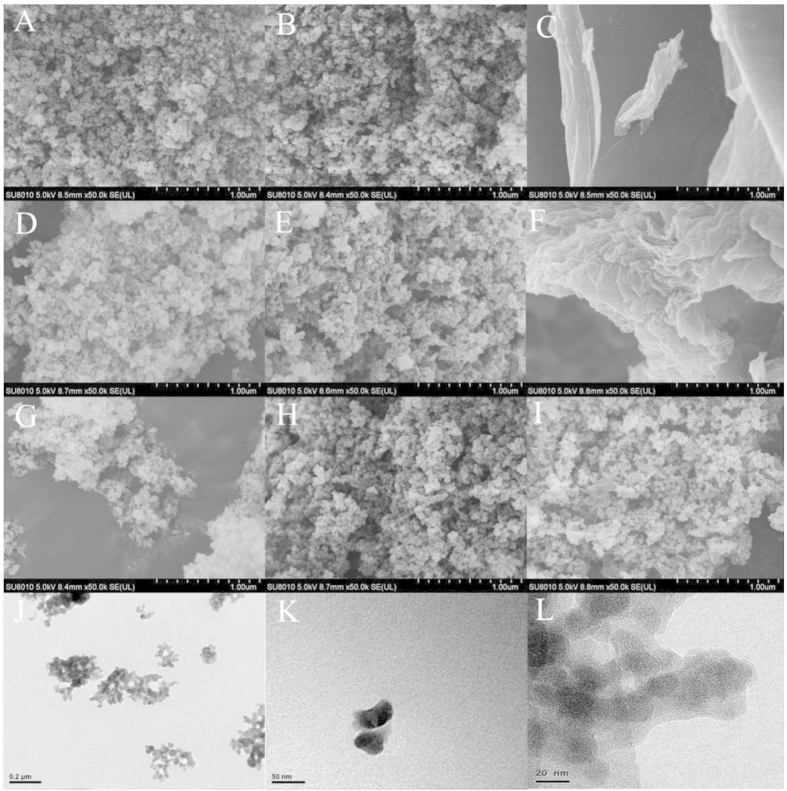
The microstructure of materials. SEM images of SiO_2_ (**A**), NH_2_-SiO_2_ (**B**), graphene oxide (**C**), ASGO (100:2) (**D**), ASrGO (100:2) (**E**), pristine graphene sheets (**F**), ASG (100:0.5) (**G**), ASG (100:1) (**H**) and ASG (100:2) (**I**). HR-TEM images of ASG (100:2) in bulk view (**J**) and at high magnification (**K**, **L**).

Based on theoretical calculation, 1 g of NH_2_-SiO_2_ (surface area = 109 m^2^/g) can support 82.9 mg of monolayer graphene (surface area = 2630 m^2^/g) at maximum degree. Four different SiO_2_-graphene ratios (100:0.5, 100:1, 100:2, and 100:4) were designed to investigate the maximum loading percentage considering the surface area reduction of NH_2_-SiO_2_ caused by aggregation. As presented on Fig. S1 in supporting information, NH_2_-SiO_2_ (positive) and graphene oxide (negative) were oppositely charged at pH 1 to 8; thus, the self-assembly of graphene oxide on NH_2_-SiO_2_ was easily performed via electrostatic interactions. The synthesis pH was controlled at 7 to maintain the maximum surface potential difference between graphene oxide and NH_2_-SiO_2_, according to surface potential analysis. The digital images presented in Fig. 1A show that the color of the precipitate became darker and the supernatant became brown gradually with increasing graphene oxide addition, indicating that more graphene oxide coated the surfaces of the SiO_2_, as well as the fact that not all of the graphene oxide completely combined with the SiO_2_. Once the loading percentage surpassed 2%, large quantities of graphene oxide were suspended in the supernatant (Fig. 1A). The graphene oxide formed large 2D planes, and multiple particles of SiO_2_ nanoparticles were enveloped by graphene oxide nanosheets during fabrication, which was evidenced by TEM images (Fig. 2J,L). The wrapped SiO_2_ particles formed an aggregation situation (Fig. 2L), which concealed a portion of the material’s surface area. Therefore, the practical fabrication did not reach its theoretical maximum loading amount.

To study the potential adsorption capacity of graphene and clarify the effects of loading percentages on its adsorption performance, nine graphene-coated materials were fabricated by loading graphene oxide onto SiO_2_ nanoparticles as a substrate at different concentrations (100:0.5, 100:1, and 100:2) and with various degrees of reduction (GO, rGO, and G). The macroscopic morphologies of the obtained GCM powders are shown in Fig. 1B. The color of the ASGO became darker as the graphene oxide loading percentage increased. After reduction, the color darkened from gray to black, and the degree of color change increased with the enhancement of reduction degree. The change in GCM color can be regarded as an expression of the reduction degree and graphene loading percentage. As tested via precipitation experiments, the graphene-coated silica materials displayed good segregation properties in aqueous solution (Fig. 1C). The GCMs naturally precipitated out of solution within 15 min, regardless of the hydrophilicity or hydrophobicity of the graphene material surfaces. Although the graphene oxide was well dispersed in the aqueous solution (Fig. 1C), it was easily precipitated and was separated from the water when SiO_2_ was added. In this case, no graphene oxide was dissolved, even after ultra-sonication (Fig. 1C). Furthermore, the graphene oxide was also stable across solution pH values between 2 and 13. Because of its strong hydrophobic surface properties and ultra-light properties, some of the pristine graphene floated on the water surface (Fig. 1C). However, once the graphene was combined with SiO_2_, all of the graphene materials precipitated, making separation much easier (Fig. 1C). The exact graphene content in the final graphene-coated materials was determined according to the variation of C content detected by elemental analysis. The graphene concentrations in ASG (100:0.5), ASG (100:1), and ASG (100:2) were 0.59%, 1.49%, and 2.36%, respectively.

The microscopic morphologies and surface structures of the graphene-based materials were observed by SEM and HR-TEM (Fig. 2). The SiO_2_ particles were circular nano-balls, and the aggregation situation created a type of microspore structure (Fig. 2A). The morphology of the SiO_2_ nanoparticles experienced little change during modification with monolayer –NH_2_ (Fig. 2B). As expected, the pristine graphene was highly aggregated and presented a blocky structure (Fig. 2F). Many wrinkles, considered to be high-energy adsorption sites, appeared on the surface of the pristine graphene. Compared with the original SiO_2_ particles, certain small extended layers appeared in the GCMs; these layers are inferred to be the graphene coating. With higher degrees of graphene loading, the extended layers became increasingly obvious (Fig. 2G–I). The extended sheets of the coated graphene were small, thin and well dispersed, rather than aggregated, and the microscopic structure exhibited almost no changes after different reduction processes (Fig. 2D,E,I). Further observations under HR-TEM (Fig. 2J–L) indicated that the graphene sheets were wrapped around the outside of the SiO_2_ nanoparticles in ultra-thin layers, suggesting that the stacked interlaminations of graphene were separated and that the process avoided strong aggregation. Obviously, the surface morphologies of the GCMs were controlled by the SiO_2_ nanoparticles rather than the graphene oxide nanosheets.

The surface area of SiO_2_, NH_2_-SiO_2_, pristine graphene and GCMs were all measured to determine the effects of combination on their surface areas (Fig. S2 in the supporting information). The surface area of pristine graphene was 277 m^2^/g, which was much lower than the theoretical surface area (2630 m^2^/g) of graphene sheets. The surface areas of GCMs largely depended on SiO_2_, when graphene was wrapped around SiO_2_ particles. There were no changes in the surface area of GCMs with different reduction processes and loading percentages. After wrapping, the loaded graphene was unfolded after being attached to the surface of the SiO_2_, which was demonstrated by the SEM and TEM investigations (Fig. 2). Based on the theoretical surface area of graphene and the experimental surface area of SiO_2_, the available support area of SiO_2_ was sufficient for monolayer graphene coverage. This finding indicates that the surfaces of the loaded graphene nanosheets were completely exposed as potential adsorption sites.

The surface bonding functional groups of GCMs were detected by FTIR, which are presented in Fig. S3 in the supporting information. In a comparison between pristine graphene and graphene oxide, the weak peaks of –OH, C=O and O-H at 3418, 1724 and 1623 cm^−1^ indicate that the reduction process eliminated many OCGs^17^. The spectra of graphene and graphene oxide displayed an extra C-H peak at 1401 cm^−1^, and the SiO_2_ and NH_2_-SiO_2_ spectra had characteristic Si-O-Si, SiO-H, and Si-OH peaks^32^. The presence of these characteristic peaks in the GCMs meant that the graphene and SiO_2_ were successfully combined. With the enhancement of reduction degree, the peak intensity of –OH at 3418 cm^−1^ decreased, suggesting that the OCGs on the surface of the loaded graphene nanosheets were reduced.

The surface potential primarily depends on the bonding functional groups. The surface charge properties of the adsorbents were investigated to better reveal potential properties for further application (Fig. S1). Before –NH_2_ modification, SiO_2_ was negatively charged, then became positively charged in the range from pH 1–8 after bonding with –NH_2_. The pristine graphene’s negative charge as OCGs could not be completely eliminated after reduction. The facile self-assembly largely altered the graphene microstructure. Based on the analyses, the surface charge of the GCMs was positive for pH values between 1 and 7. This positive charge was controlled by the NH_2_-SiO_2_ particles inside of the composite rather than the graphene ad-layer on the outside and exhibited no change with variations in the loading percentages or reduction degrees.

### Adsorption isotherms of the graphene-coated materials

**Figure 3:**
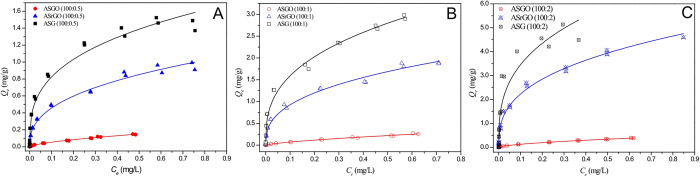
Adsorption isotherms of phenanthrene on the graphene-coated materials, including graphene oxide (ASGO), reduced graphene oxide (ASrGO), and graphene (ASG), at different loading rates.

The adsorption isotherms of phenanthrene on nine different GCMs are shown in Fig. 3. The isotherm of pristine graphene and ASG(100:0.5) are cited from our previous study^19^ for comparison. The regression parameters of the isotherms obtained according to the Freundlich model are listed in Table 1. The Freundlich equation is an empirical model based on adsorption onto a heterogeneous surface^17^,^33^,^34^ and is described as follows:


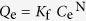


where *K*_f_ [(mg/g)/(mg/L)^N^] is the Freundlich affinity coefficient, *N* is the exponential coefficient, *Q*_e_ (mg/g) is the equilibrium adsorption concentration of the sorbent, and *C*_e_ (mg/L) is the equilibrium solution phase concentration of the sorbate. According to the regression coefficient (R^2^), the isotherms of the nine different graphene-based materials were well fitted by the Freundlich model. The Freundlich *N* index was 0.698−0.612 for ASGO at 100:0.5, 100:1 and 100:2, and decreased to 0.387−0.324 for ASrGO and ASG, suggesting that the surfaces of the ASrGO and ASG were more heterogeneous than those of ASGO. The adsorption of GCMs increased as the number of OCGs on the graphene nanosheets decreased (Fig. 3) and increased as the loading percentage of the graphene materials increased (Fig. S4). These observations suggest that the loaded graphene nanosheets dominated the adsorption capabilities of the GCMs.

According to the loading percentage test, the maximum graphene loading degree is 2% under which graphene oxide can mostly be loaded onto the surface of NH_2_-SiO_2_. Based on Fig. 3C and Table 1, the overall phenanthrene adsorption capacity (*Q*_e_) at *C*_e_/*C*_s_ = 0.01 was 3.62 × 10^−2^ mg/g for ASGO (100:2) which was approximately 660 times higher than that of SiO_2_ (5.44 × 10^−5^ mg/g)^19^. When ASGO (100:2) was treated by solvothermal reduction, the *Q*_e_ at *C*_e_/*C*_s_ = 0.01 for ASrGO (100:2) further increased to 1.08 mg/g and up to 1.74 mg/g for ASG (100:2) after deeper chemical reduction. The adsorption capacity of ASG (100:2) was approximately 32,000 times greater than that of bare SiO_2_. Meanwhile, the exact graphene content in ASG (100:2) was only 2.35% according to the elemental analysis. Considering the widespread existence of SiO_2_ in the environment and the mass production of graphene, the combination of graphene and SiO_2_ is unavoidable. Companied with graphene’s commercial process, this dramatic improvement in the adsorption of SiO_2_ should increase our focus on controlling graphene as an environmental risk. Similarly, the adsorption capacity of GCMs with loading percentages of 100:1 and 100:0.5 (Fig. 3B,C) increased with enhanced reduction. As presented in Table 1, the *Q*_e_ of pristine graphene (2.45 mg/g at *C*_e_/*C*_s_ = 0.01)^19^ was clearly greater than that of these graphene-coated materials because the graphene content in the GCMs was minimal. Neglecting the minimal affinity of SiO_2_ with phenanthrene, the adsorption capacity of graphene in ASG (100:2) was 73.73 mg/g, which was 30 times greater than that of pristine graphene (2.45 mg/g) (based on the same graphene content at *C*_e_/*C*_s_ = 0.01). This surprising increase indicated that the potential adsorption capacity of the well-dispersed graphene was successfully exposed in higher graphene loading percentage and that the results were higher than the relevant data presented in previous reports^17^,^23^,^35^.

The combination of graphene and SiO_2_ produced a synergistic effect in aromatic pollutant removal, and the large-scale improvements in the adsorption of graphene suggested that wrapping graphene around SiO_2_ particles is a facile method for using graphene to manage environmental pollution. In addition to the degree of reduction, the influence of the graphene loading percentage on the adsorption performance was also investigated (Fig. S4). The *Q*_e_ values of ASG (100:0.5), ASG (100:1), and ASG (100:2) were 0.424, 0.775, and 1.74 mg/g, respectively, at *C*_e_*/C*_s_ = 0.01. When normalized by the graphene contents of the GCMs, the adsorption of phenanthrene onto wrapped graphene in ASG (100:0.5), ASG (100:1) and ASG (100:2) were 71.86, 52.01 and 73.73 mg/g, respectively, at *C*_e_*/C*_s_ = 0.01. This excellent adsorption performance illustrates that the potential adsorption ability of the graphene nanosheets was readily available after the graphene was applied to silica particles under the loading ratio below 2%. Because the adsorption performance of the GCMs was influenced by the synthesis conditions, the ratio of graphene to SiO_2_, and the oxygen content in the graphene surfaces, this study provides a reference for fabrication parameters in designing optimal graphene-coated materials for potential environmental applications.

### Concentration-dependent *K*_d_/*K*_HW_ of phenanthrene on GCMs

**Figure 4:**
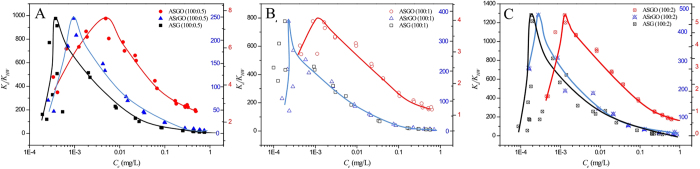
Concentration-dependent *K*_d_/*K*_HW_ values of phenanthrene on graphene-coated materials with different loadings and reduction degrees (**A**–**C**). The y-axis scale is presented on the left side for ASG, on the right inside scale for ASrGO, and the right outside scale for ASGO.

To further analyze the adsorption capability of graphene nanosheets in the GCMs, the distribution coefficient (*K*_d_ = *Q*_e_/C_e_) was introduced. To better exhibit the mutual interaction mechanism beyond the hydrophobic effect, the normalized adsorption coefficient (*K*_d_) relative to the hexadecane-water partitioning coefficient of phenanthrene (*K*_HW_ = 35481 L/kg) was also applied^17^,^35^. The concentration-dependent *K*_d_/*K*_HW_ values of phenanthrene adsorption on the GCMs are presented in Fig. 4, noting that the *K*_d_/*K*_HW_ values of phenanthrene adsorption on ASG (200:0.5) was cited from our previous study for comparison^19^. Interestingly, for all of the nine GCM, the *K*_d_/*K*_HW_ ratio increased with the sorbate equilibrium concentration and formed a sharp peak at relatively low concentration ranges. This uncommon phenomenon for the adsorption of GCMs occurred by varying the concentrations of the sorbate over 3 orders of magnitude rather than over a narrow concentration range. The increase in *K*_d_/*K*_HW_ with increasing sorbate concentration suggests that greater surface energy adsorption sites were created on the graphene nanosheets during the initial adsorption stage. With more phenanthrene attached to the graphene surface, the original and subsequently created sites were gradually occupied, resulting in a decline in the *K*_d_/*K*_HW_. A sharp peak in *K*_d_/*K*_HW_ appeared for all ASGO, ASrGO, and ASG samples. The turning point clearly shifted to lower equilibrium concentrations from ASGO, to ASrGO, and further to ASG, with enhanced reduction degree (Fig. 4A–C).

**Figure 5:**
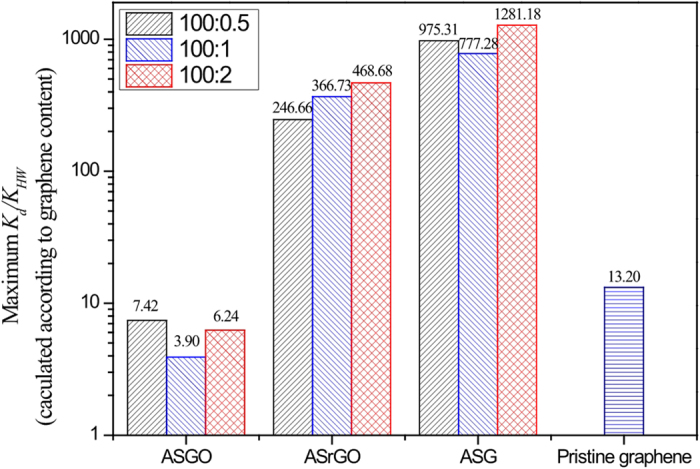
Comparison of the maximum *K*_d_/*K*_HW_ values of phenanthrene on pristine graphene and graphene-coated materials with different loadings and reduction degrees. The amount of adsorption was normalized to the graphene content.

Based on Fig. 4, the magnitude of the *K*_d_/*K*_HW_ values for graphene material decreased as follows ASG >ASrGO >> pristine graphene (maximum *K*_d_/*K*_HW_ = 13)^19^ > ASGO. To illustrate the excellent affinities of the graphene-coated materials, the maximum *K*_d_/*K*_HW_ values based on the graphene contents of different GCMs are compared in Fig. 5. The *K*_d_/*K*_HW_ values of phenanthrene on ASrGO (100:2) and ASG (100:2) were approximately 75 and 205 times greater, respectively, than that of ASGO (100:2). The maximum *K*_d_/*K*_HW_ ratio of graphene in ASG (100:2) was 1280, which was approximately 100 times greater than that of pristine graphene. However, the enrichment capacity of loaded graphene with regard to aromatic molecules was almost 1000 times higher than biological interfaces, which can be determined because *K*_HW_ is widely used as a surrogate to simulate organic molecule partitioning on biological interfaces^36^. The affinity of loaded graphene did not decrease with increasing graphene addition, indicating that the graphene is well dispersed at high loading ratios. This result suggests that the graphene-coated materials function as superior adsorbents for nonpolar aromatic pollutants. The maximum *K*_d_/*K*_HW_ (1280) for phenanthrene on ASG was much higher than for other popular adsorbents, such as single-walled carbon nanotubes (maximum *K*_d_/*K*_HW_ = 280 for phenanthrene)^37^, activated carbon (maximum *K*_d_/*K*_HW_ = 0.493)^38^, coke (maximum *K*_d_/*K*_HW_  = 1.13 × 10^−3^)^38^, silica (maximum *K*_d_/*K*_HW_ = 1.83 × 10^−4^)^39^, alumina (maximum *K*_d_/*K*_HW_ = 1.13 × 10^−3^)^39^ and modified bentonite (maximum *K*_d_/*K*_HW_ = 0.564 and 0.324–1.27)^40^,^41^. Therefore, the graphene nanosheets anchored to the SiO_2_ nanoparticles acted as a superior sorbent for aromatic pollutant removal.

### Interaction mechanisms of graphene-based materials with phenanthrene

As a well-studied nanomaterial, SiO_2_ nanoparticles are known to possess a hydrophilic surface with a large quantity of –OH^32^,^39^. The hydrophilic groups interact with water molecules through hydrogen bonds, and the surfaces of the SiO_2_ nanoparticles are naturally covered by a hydration shell^30^,^31^,^42^. During the adsorption process, this hydration shell acts as a barrier that limits the transfer of phenanthrene (hydrophobic compound) from bulk water to the SiO_2_ surface, the adsorption of phenanthrene onto SiO_2_ in the aqueous solution was minimal. The Freundlich *N* index of SiO_2_ (1.06)^19^ approached one, indicating that the interaction mechanisms between SiO_2_ and phenanthrene primarily resulted from the distribution effect^39^. The adsorption of phenanthrene onto SiO_2_ involved its transfer from the bulk liquid phase to the vicinal water phase^39^. After modification by APTES, the adsorption of SiO_2_ showed little change because the bound –NH_2_ was a monolayer and multiple –OH groups were preserved^43^. Interestingly, after a small amount of graphene layer was added to the surface of the SiO_2_ particles, the adsorption capacity of the GCMs significantly improved. This improvement was mainly attributed to the existence of graphene nanosheets acting as a hydrophobic cloth. Once wrapped, the hydrophilic surface of the SiO_2_ particles became hydrophobic and possessed an additional π-electron conjugated structure. The strong π-π interaction between the phenanthrene and graphene nanosheets was accompanied by a hydrophobic interaction, replacing the weak distribution behavior in the vicinal water phase. Consequently, the GCMs displayed an excellent affinity for phenanthrene.

In addition to large improvements in the adsorption capacities of the SiO_2_ particles, the adsorption capacities of the coated graphene on the SiO_2_ significantly improved relative to pristine graphene at the same concentration. After wrapping around the surface of the SiO_2_ particles, the microscopic morphology of graphene was loose and porous (Fig. 2). The stacked interlamination in the pristine graphene was opened, and the nanosheets were exfoliated by adding SiO_2_^19^, which made it possible for the graphene layers to fully express their potential adsorption sites. Furthermore, the surface charges of the graphene-based materials were largely dependent on NH_2_-SiO_2_, which was positively charged (Fig. S1 in the supporting information). Additional electrostatic repulsion caused the graphene-based materials to unfold and expose adsorption sites during the adsorption process.

Because phenanthrene has no additional polar group, its main interaction mechanism with graphene is considered to be hydrophobic interactions and involves π-π interactions between the graphene nanosheets and phenanthrene^17^,^18^,^20^. According to classical interaction mechanisms, phenanthrene should preferentially occupy adsorption sites with high surface energies, which include defects, edges and wrinkles^17^,^20^,^21^,^23^,^44^. During the adsorption process, the affinity between phenanthrene and graphene should decrease after the sites with high surface energies are occupied, resulting in a decrease in the *K*_d_/*K*_HW_ value. As shown in Fig. 4, the decrease in the *K*_d_/*K*_HW_ ratio after the peak is consistent with this speculation. However, an unexpected increase in *K*_d_/*K*_HW_ values was observed in the adsorption process of phenanthrene on graphene at relatively low concentrations, and the peaks were obviously shifted to lower phenanthrene concentrations. This unusual behavior cannot be explained by the hydrophobic effect and π-π interactions alone, indicating that additional interaction mechanisms were involved at the molecular graphene-water interface.

**Figure 6:**
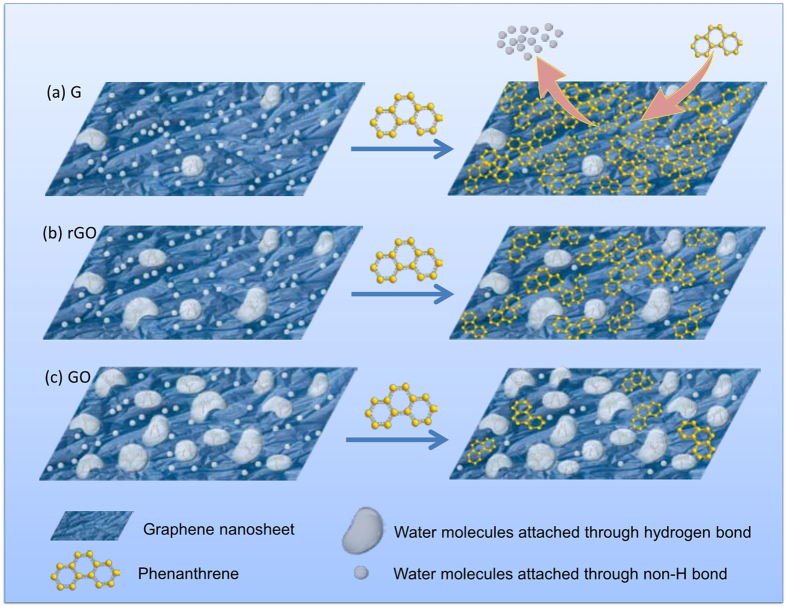
Schematic images of the states of water molecules on graphene with different surface and their influences on phenanthrene adsorption. (**a**) On the plane of graphene nanosheets. (**b**) On the plane of reduced graphene oxide. (**c**) On the plane of graphene oxide. Note that the water microdroplets formed via non-H bonds can be replaced by phenanthrene molecules, which contributed to the excellent adsorption of graphene nanosheets besides the hydrophobic effect and π -π interactions.

In aqueous solutions, water molecules are unavoidable adsorbates on hydrophobic surfaces of graphene nanosheets^45^. As previously reported, water molecules exist on the surface of graphene in two forms, a hydration shell (water film) aggregated around OCGs through strong hydrogen bonds and water microdroplets attached to hydrophobic graphene coupled by weak non-H bonds (Fig. 6), which are structurally and dynamically different from those of the bulk liquid^45^,^46^,^47^. Experimental data and theoretical calculations suggest that H_2_O adsorbed via weak non-H bonds can act as a p-type molecular dopant on the graphene surface and as a charge acceptor to induce an increase in the concentrations of graphene charge carriers (holes)^48^,^49^. Therefore, the charge transfer interaction is the main contributor to water microdroplets or nanodroplets on graphene surfaces. The microscopic structure of this coupled water is ice-like, with a highly oriented array^45^,^47^,^50^, which partly indicated that such water microdroplets are stable on the surface of graphene nanosheets in water. In combination with a defective SiO_2_ substrate, both highly oriented water clusters and water adsorbates can lead to graphene doping^46^. The distinct character of attached water may generate uncommon interactions on the graphene-water interface.

The hydrated shell functions as a hydrophilic barrier to weaken the hydrophobic character of the graphene and further prevents π-π interactions between the phenanthrene and the graphene oxide surface (Fig. 6C). Therefore, the adsorption capacity of graphene oxide is much weaker than that of graphene^17^. Reduction is the efficient way to modulate the hydrated shell by decrease OCGs quantity. The distribution of non-H-bond water microdroplets is closely related to the morphology of the graphene. These microdroplets preferentially occupy sites with high surface energies, such as defects, edges and wrinkles. In addition, the existence of these water microdroplets enhances the hydrophilic character of the graphene^45^. Presumably, the water microdroplets formed via weak non-H bonds can easily be replaced by phenanthrene molecules through strong π-π interactions (Fig. 6A). Recently, the study of Apul *et al*. also suggested that aromatic molecules may be better aligned on graphene sheets^35^. This replacement corresponds with increasing entropy. Because phenanthrene is a hydrophobic, aromatic, and flat compound, this replacement enables the recovery of the hydrophobic character of the graphene that has been doped by coupled water microdroplets. Compared with flat surfaces, more replacement may occur on the defects, edges and wrinkles of the graphene, which are considered to be high surface energy sites for phenanthrene adsorption^17^. Phenanthrene may act in a patch-like fashion to mend graphene defects via aromatic benzene. After replacement, the hydrophobic character of the graphene is strengthened, resulting in a higher total free energy. To maintain self-stability, more wrinkles could be created intrinsically^51^. This is likely the primary reason why the addition of phenanthrene changes the morphology of graphene^17^. The micro-replacement mechanisms of water microdroplets or nanodroplets by phenanthrene precisely agree with the increase in *K*_d_/*K*_HW_ values at low concentration ranges and may provide new insights into the phenanthrene adsorption mechanisms at the graphene-water interface.

The distributions of water clusters and water microdroplets/nanodroplets are regulated by oxygen-containing groups, which are influenced by the degree of reduction of the graphene-coated materials. During the reduction process, the π electron structure and hydrophobic surface of the graphene are recovered^17^,^50^,^52^. Consequently, the adsorption capacity of the graphene-based materials showed considerable improvement. The elimination of OCGs makes it difficult for water molecules to aggregate around OCGs though hydrogen bonds, which results in the production of a less-hydrated shell. Correspondingly, the surface of graphene became more available for water microdroplets rather than water clusters/films (Fig. 6). In this situation, the non-H-bond water microdroplets in the graphene nanosheets were more efficiently replaced by phenanthrene molecules. This result may explain why the sorbate concentration at the peak *K*_d_/*K*_HW_ values shifted to lower values from ASGO to ASrGO and ASG.

## Conclusion

The substantial aggregation of graphene nanosheets can be easily overcome via the coating of SiO_2_ particles, which then act as a framework. The potential adsorption sites concealed in stacked graphene were exposed in the GCMs, strongly enhancing the adsorption of the graphene nanosheets. Through surface potential manipulation, the potential adsorption capacity can be well expressed at its maximum loading degree (2%). Additionally, the separation of hydrophobic and hydrophilic graphene materials from water improved when SiO_2_ particles were used. The water molecules (microdroplets) attached to the exposed graphene nanosheets via non-H bonds are easily replaced by phenanthrene molecules, and the displacement efficiency can be regulated by oxygen-containing groups of graphene nanosheets. The graphene nanosheets and silica particles produce a synergetic effect in pollutant-removing GCMs, presenting a facile method for large-scale production of graphene-coated materials. Graphene-coated materials using silica particles as a framework are cost-effective and highly efficient adsorbents for removing aromatic pollutants from water and will result in enormous opportunities for using novel 2D nanomaterials (graphene) for environmental applications.

## Methods

### Preparation of graphene-coated materials

Graphene oxide (GO) was prepared from natural graphite flakes using a modified version of the Hummers method^53^. The SiO_2_ nanoparticles were first modified using a silane coupling agent (APTES) to transform the surface potential by binding monolayer –NH_2_^46^. The detailed fabrication processes are presented in the Supporting Information. First, 1 g of modified SiO_2_ particles were dispersed in deionized water (50 mL) by stirring. A selected concentration of graphene oxide solution (50 mL) was prepared using ultrasonication. Subsequently, the graphene oxide solution was gradually added to the mixed NH_2_-SiO_2_ colloids using continuous mechanical stirring. The whole process was kept at 35 °C for 1 h, and the final pH was controlled at 7. After completion of the reaction, the mixture was undisturbed for 2 h to observe the settling situation. The final aminosilica-supported graphene oxide (ASGO) was collected through filtration, washed with water and dried under vacuum. The loading percentage of graphene was controlled by modulating the initial concentration of the graphene oxide solution and was determined by elemental analysis. ASGO (100:0.5) was synthesized by adjusting the initial concentration of the GO solution to 0.1 mg/mL to obtain the SiO_2_-to-GO ratio of 100:0.5. GO solutions of 0.2 mg/mL, 0.4 mg/mL and 0.8 mg/mL were used for ASGO (100:1), ASGO (100:2) and ASGO (100:4), respectively.

Solvothermal reduction and chemical reduction were used to deoxygenate of graphene oxide in the ASGO to prepare the reduced graphene oxide (rGO) and graphene (G) loading on SiO_2_, respectively. Compared with solvothermal reduction, the degree of deoxygenation via chemical reduction was much higher^50^. The chemical reduction procedure proceeded as follows: ASGO (100 mg) was added to water (100 mL) while stirring. Then, a selected volume of hydrazine hydrate was injected. After placing the mixture in a water bath (98 °C) for 24 h, the final reduced product was collected through filtration and washed multiple times with water before vacuum drying. The solvothermal reduction was performed in a 100-mL polytetrafluoroethylene-lined stainless-steel autoclave. After maintaining the temperature at 150 °C for 10 h, the product was collected through filtration and vacuum dried. The products of the chemical reduction and solvothermal reduction processes were identified as ASG and ASrGO, respectively.

### Structural characterization

The surface areas of the GCMs were measured using N_2_ adsorption-desorption at –196 °C with a NOVA-2000E surface area analyzer and were calculated using the multipoint Brunauer-Emmett-Teller (BET) method. The exact concentrations of C, H, and N were determined using an EA112 CHN elemental analyzer (Thermo Finnigan). The surface charge properties were evaluated at different equilibrium pH values using a Malven Zetasizer analyzer. The surface functional groups were monitored with a Bruker Vector 22 FTIR spectrometer at a resolution of 1 cm^−1^ using a mixture of 1 mg graphene-coated materials and 200 mg KBr. The surface morphologies and structures were characterized using an S-4800 field-emission scanning electron microscope (FE-SEM) (Hitachi, Tokyo) and a high-resolution transmission electron microscope (HR-TEM) (JEOL, Japan). For the FE-SEM observations, samples were placed on the conductive base without any extra treatment to detect the actual morphologies and gold nano-film was sprayed for better conductivity. For the HR-TEM observations, the samples were dispersed in a water solution to yield individual particles and dropped onto the micro grid network.

### Batch adsorption experiment

Phenanthrene was selected as the model aromatic pollutant to explore the adsorption capacity of GCMs with different loadings and various oxygen contents. Adsorption experiments were conducted at room temperature (25 ± 1 °C) in a background solution (with pH = 7 and containing 0.01 mol/L CaCl_2_ and 200 mg/L NaN_3_ to control the ionic strength and inhibit microbial activity). For any given GCM, the adsorption capacities obtained after 36 h and 72 h were the same, suggesting that equilibration was achieved before 36 h. Isothermal experiments with different materials were conducted at selected water-to-solid ratios to maintain removal percentages of 20%–80%. Different volumes of phenanthrene in acetonitrile were dissolved in the background solution to achieve the desired concentration. The volume of acetonitrile in the background solution was maintained at less than 0.1% to avoid cosolvent effects^17^. After shaking the suspension in the dark at 120 rpm for 36 h to reach final equilibrium, the solution was separated from the solid by centrifugation and the final concentration of phenanthrene in the supernatant solution was determined by high-performance liquid chromatography. For comparison, phenanthrene isotherms of ASG (100:0.5) and pristine graphene were cited from our previous study^19^.

## Additional Information

**How to cite this article**: Yang, K. *et al*. Graphene-coated materials using silica particles as a framework for highly efficient removal of aromatic pollutants in water. *Sci. Rep*. **5**, 11641; doi: 10.1038/srep11641 (2015).

## Supplementary Material

Supplementary Information

## Figures and Tables

**Table 1:** Freundlich model regression parameters of adsorption isotherms of phenanthrene onto SiO_2_, NH_2_-SiO_2_, pristine graphene, and nine graphene-coated materials of graphene oxide (ASGO), reduced graphene oxide (ASrGO), and graphene (ASG) with different loadings of graphene.

Adsorbent	*K*_f_	*N*	R^2^	*Q*_e_, mg/g	*K*_d_, L/g
				at *C*_e_/C_s_ = 0.01	
pristine graphene^19^	149.26 ± 20.24	0.945 ± 0.072	0.967	2.45	191
ASGO(100:0.5)	0.2363 ± 0.005F0	0.646 ± 0.016	0.996	0.0142	1.14
ASrGO(100:0.5)	1.1138 ± 0.0246	0.387 ± 0.017	0.987	0.207	16.9
ASG(100:0.5)^19^	1.7371 ± 0.0505	0.324 ± 0.019	0.976	0.424	34.9
ASGO(100:1)	0.3571 ± 0.0077	0.698 ± 0.022	0.995	0.0171	1.37
ASrGO(100:1)	2.1852 ± 0.0458	0.373 ± 0.015	0.989	0.431	35.3
ASG(100:1)	3.5684 ± 0.0814	0.351 ± 0.014	0.990	0.775	63.6
ASGO(100:2)	0.5192 ± 0.0049	0.612 ± 0.009	0.999	0.0362	2.90
ASrGO(100:2)	5.0375 ± 0.1295	0.354 ± 0.016	0.987	1.08	88.6
ASG(100:2)	7.4338 ± 0.5540	0.333 ± 0.031	0.928	1.74	143
