# Supplementary material for: Graphene-coated materials using silica particles as a framework for highly efficient removal of aromatic pollutants in water
Source: Sci Rep. 2015 Jun 29;5:11641. doi: 10.1038/srep11641 (PMC4484242; doi:10.1038/srep11641)
Supplement: Supplementary Information [file srep11641-s1.pdf]

Appendix A. Supplementary data for

Scientific Reports

Graphene-coated materials using silica particles as a framework for highly efficient removal of aromatic pollutants in water

Kaijie Yang[1,2]; BaoLiang Chen[1,2*]; Lizhong Zhu[1, 2]

1. Department of Environmental Science, Zhejiang University, Hangzhou 310058, China;

2. Zhejiang Provincial Key Laboratory of Organic Pollution Process and Control, Hangzhou 310058, China;

*Corresponding Author Phone & Fax: 86-571-88982587

Email: blchen@zju.edu.cn

Supporting Information consists of 5 pages, including this one.

There are 4 Figures.

Contents:

Synthesis of graphene oxide. `S2

Synthesis of amino-silica (NH_2-SiO_2). S2

Fabrication of GCMs with different graphene loading percentage and reductive degree. S3

Figure S1. Zeta potential curves of NH_2-SiO_2, GO and GCMs at different pH values. S4

Figure S2. Comparison of surface area between SiO_2, NH_2-SiO_2, pristine graphene and GCMs. S4

Figure S3. FTIR spectra of ASGO (5200:1), ASrGO (5200:1), ASG (5200:1), SiO_2, NH_2-SiO_2, GO and pristine graphene. S5

Figure S4. Adsorption isotherms of phenanthrene on ASG (A), ASrGO (B), and ASGO (C) with different loading percentages. S5

Synthesis of graphene oxide. Graphene oxide (GO) was synthesized from natural graphite flakes using a modified version of the Hummers method. The detailed procedures are described as follows. To avoid graphite-core/GO-shell particles, the graphite was pre-oxidized. After adding 8.33 g of $K_2S_2O_8$ and 8.33 g of P_2O_5 into 30 mL of concentrated H_2SO_4, 10 g of graphite flakes were added and the mixture was maintained at 80 ºC for 4.5 h. Next, the product was collected through filtration and washed until the pH became neutral. After vacuum drying, the pre-oxidized graphite was obtained.

The concentrated H_2SO_4 was pre-cooled in an ice bath. Next, 10 g of pre-oxidized graphite, 5 g of $NaNO_3$, and 30 g of $KMnO_4$ were added to the pre-cooled H_2SO_4 solution while stirring. After stirring for 2 h, 460 mL of deionized water was added to heat the mixture and increase the level of oxidation. Before obtaining the GO, 22 mL of 30% H_2O_2 was added for further oxidation. After centrifugation, the solid phase was washed with 10% HCl to remove the residual SO_4^- and metal ions, and then the solid phase was redispersed in water and peeled by ultrasonication for 30 min. The centrifugation and sonication steps were repeated 3 times to completely peel the graphene nanosheets. To remove other impurities, the final solution of GO was subjected to dialysis until it reached pH 7.

Synthesis of amino-silica (NH_2-SiO_2). The surface modification of SiO_2 was performed in aqueous solution using a simple one-step synthesis. The SiO_2 (10 g) was well dispersed in ethyl alcohol (300 mL) under ultrasonication and mechanical stirring. Then, the silane coupling agent APTES (1 mL) was added to bind -NH_2. The reaction was performed at 50 ºC for 12 h to obtain modified monolayers and positively charged NH_2-SiO_2. The product was collected by filtration, washed with alcohol and water several times and dried under vacuum at 60 ºC.

Fabication of GCMs with different graphene loading percentage and reductive degree. The whole process to fabricate ASGO was processed under water solution. Firstly, 2 g NH_2-SiO_2 was completely dispersed in water solution using mechanical stirring and ultrasonication. Then 0.4 mg/ml graphene oxide solution which was well disrupted added into the NH_2-SiO_2 mixture under mechanical stirring. Reaction was controlled at 35 ºC at the final pH=7. The final product, ASGO(50:1), was collected through filtration and dried in vacuum at 60 ºC. As the same process, ASGO(100:1) and SGO(200:1) was synthesized by modulate the initial concentration of graphene oxide.

To obtain ASrGO, 280 mg ASGO was dispersed in 80 ml water and sealed in polytetrafluoroethylene. Then the reactor was putted at 150 ºC for 10h. The GCMs with deeper reduction (termed ASG) was treated by hydrazine. 100mg ASGO was dispersed in 100ml water then added proper hydrazine. The reaction was processed under 98 ºC and the amount of hydrazine was determined according to GO: hydrazine=1 mg:1 ml.

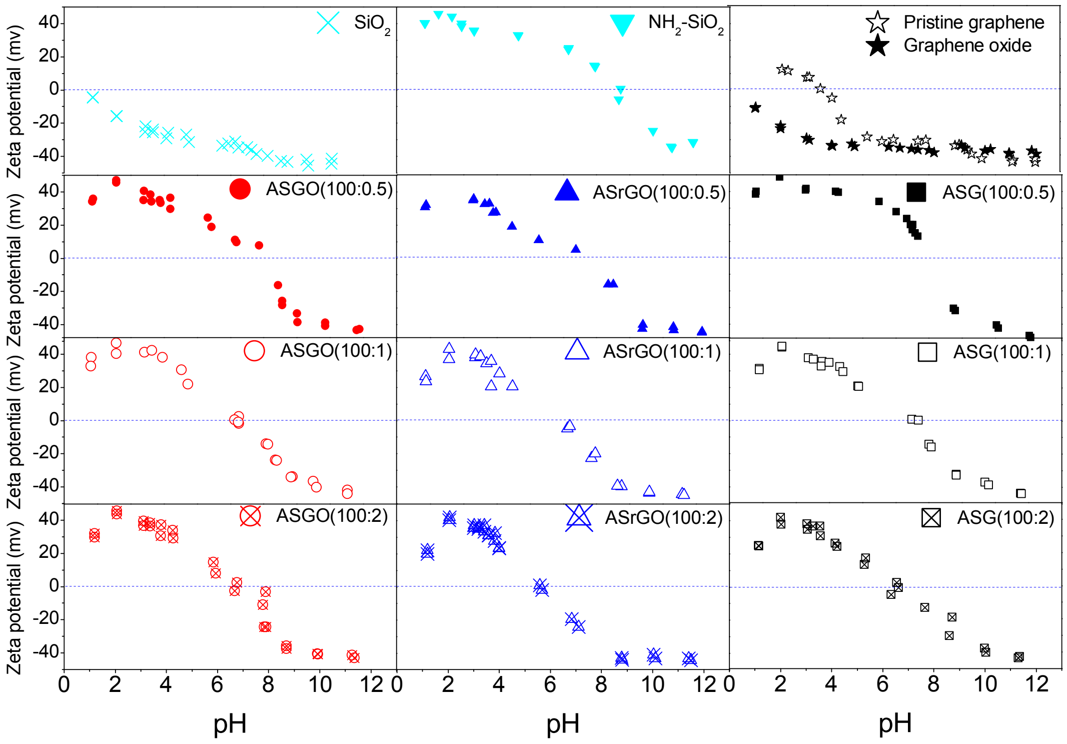

Figure S1. Zeta potential curves of NH_2-SiO_2, GO, pristine graphene and GCMs at different pH values.

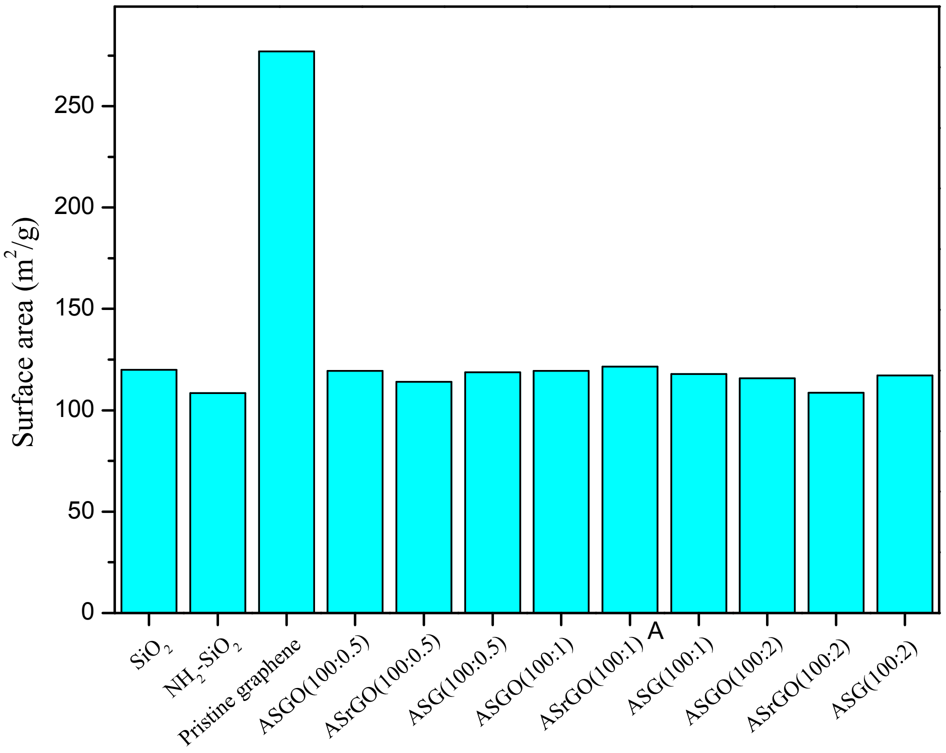

Figure S2. Comparison of surface area between SiO_2, NH_2-SiO_2, pristine graphene and GCMs.

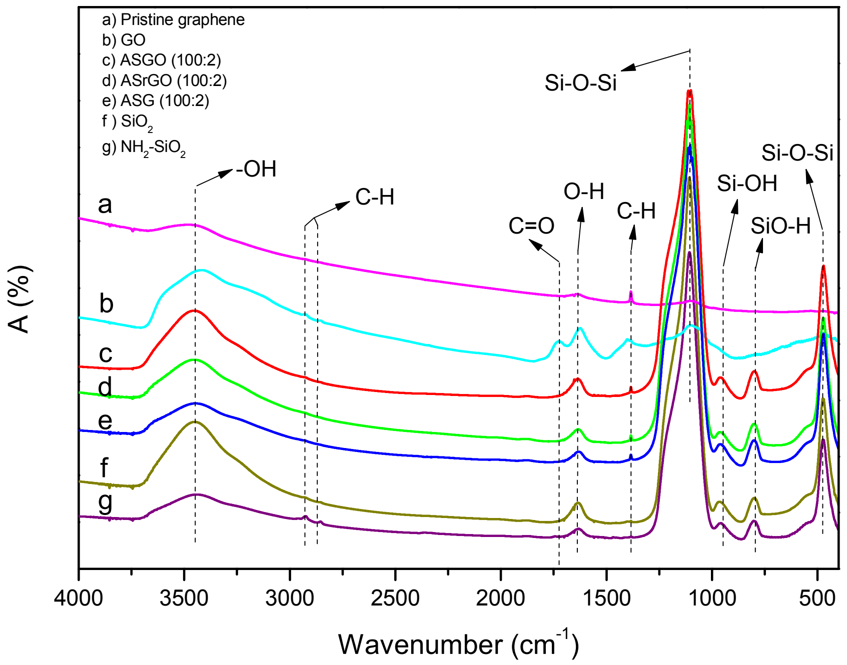

Figure S3. FTIR spectra of ASGO (100:2), ASrGO (100:2), ASG (100:2), SiO_2, NH_2-SiO_2, GO and pristine graphene.

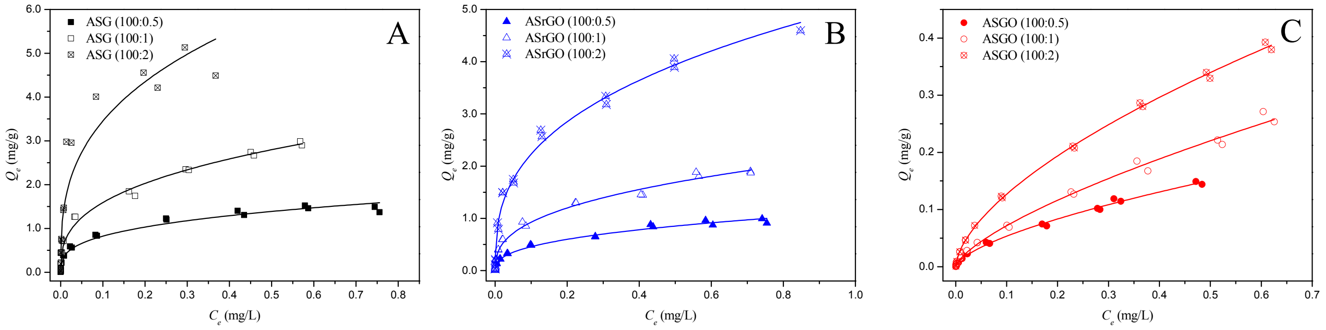

Figure S4. Adsorption isotherms of phenanthrene on ASG (A), AsrGO (B), and ASGO (C) with different loading percentages.
